# Reduced hippocampal subfield volumes and memory performance in preterm children with and without germinal matrix-intraventricular hemorrhage

**DOI:** 10.1038/s41598-021-81802-7

**Published:** 2021-01-28

**Authors:** Lexuri Fernández de Gamarra-Oca, Leire Zubiaurre-Elorza, Carme Junqué, Elisabeth Solana, Sara Soria-Pastor, Élida Vázquez, Ignacio Delgado, Alfons Macaya, Natalia Ojeda, Maria A. Poca

**Affiliations:** 1 Department of Methods and Experimental Psychology, Faculty of Psychology and Education, University of Deusto, Bilbao, Basque Country, Spain; 2 Medical Psychology Unit, Department of Medicine, Institute of Neuroscience, University of Barcelona, Barcelona, Catalonia Spain; 3 Biomedical Research Networking Center on Neurodegenerative Diseases (CIBERNED), Hospital Clinic, Barcelona, Catalonia Spain; 4 Institute of Biomedical Research August Pi I Sunyer (IDIBAPS), Barcelona, Catalonia Spain; 5 Center of Neuroimmunology, Laboratory of Advanced Imaging in Neuroimmunological Diseases, Hospital Clinic, Barcelona, Catalonia Spain; 6 Department of Psychiatry, Consorci Sanitari del Maresme, Hospital of Mataró, Mataró, Catalonia Spain; 7 Department of Pediatric Radiology, Vall d’Hebron University Hospital, Autonomous University of Barcelona, Barcelona, Catalonia Spain; 8 Grup de Recerca en Neurologia Pediàtrica, Vall d’Hebron Institut de Recerca, Hospital Universitari Vall d’Hebron, Passeig Vall d’Hebron 119-129, 08035 Barcelona, Catalonia Spain; 9 Department of Neurosurgery and Neurotraumatology and Neurosurgery Research Unit, Vall d’Hebron Research Institute, Autonomous University of Barcelona, Barcelona, Catalonia Spain

**Keywords:** Brain, Paediatric research

## Abstract

Preterm newborns with germinal matrix-intraventricular hemorrhage (GM-IVH) are at a higher risk of evidencing neurodevelopmental alterations. Present study aimed to explore the long-term effects that GM-IVH have on hippocampal subfields, and their correlates with memory. The sample consisted of 58 participants, including 36 preterm-born (16 with GM-IVH and 20 without neonatal brain injury), and 22 full-term children aged between 6 and 15 years old. All participants underwent a cognitive assessment and magnetic resonance imaging study. GM-IVH children evidenced lower scores in Full Intelligence Quotient and memory measures compared to their low-risk preterm and full-term peers. High-risk preterm children with GM-IVH evidenced significantly lower total hippocampal volumes bilaterally and hippocampal subfield volumes compared to both low-risk preterm and full-term groups. Finally, significant positive correlations between memory and hippocampal subfield volumes were only found in preterm participants together; memory and the right CA-field correlation remained significant after Bonferroni correction was applied (*p* = .002). In conclusion, memory alterations and both global and regional volumetric reductions in the hippocampus were found to be specifically related to a preterm sample with GM-IVH. Nevertheless, results also suggest that prematurity per se has a long-lasting impact on the association between the right CA-field volume and memory during childhood.

## Introduction

Preterm birth has been defined as any delivery before 37 completed weeks of gestation. According to its clinical manifestations, prematurity can be classified into two different conditions: low-risk and high-risk prematurity. Preterm newborns considered to be at a lower risk of developing neurodevelopmental alterations commonly evidence a gestational age (GA) of between 30 to 36 weeks, minor perinatal complications, and lack of brain abnormalities observed by cranial ultrasound (US)^1,2^. However, while these children are at a lower risk of presenting developmental complications, their immature nervous system might account for long-term neurodevelopmental alterations^3^. In contrast, high-risk prematurity commonly refers to preterm birth together with neonatal brain injury, which may cause long-lasting adverse complications. For instance, germinal matrix-intraventricular hemorrhage (GM-IVH) has been related to poorer overall development during infancy^4^.

Early detection of subtle or severe neonatal brain alterations may alleviate some of the neurodevelopmental consequences related to prematurity via the development of more targeted early intervention programs in order to improve the quality of life of this population. For its part, Magnetic Resonance Imaging (MRI) can accurately identify and quantitatively assess gray matter (GM) and white matter (WM) abnormalities^5^, which correlate with cognitive measures in both low- and high-risk preterm samples^6,7^. Whereas prematurity has been associated with abnormal growth in several brain regions^8^, temporal lobe alteration might occur to a greater extent than in other regions, since reduced GM is often attributed to bilateral temporal lobe damage^9^. Even in low-risk preterm children, abnormalities have been found in the superior temporal sulcus surface^10^, as well as neurochemical anomalies in adolescence^11^. Moreover, smaller hippocampal volumes are often found in high-risk preterm samples^12^. This allocortical structure that is localized in the temporal lobe appears to be particularly susceptible to adverse pre-perinatal events such as hypoxia/ischemia, which limit its connections^13,14^. Overall hippocampal volumetric reductions seem to have an impact on memory performance in prematurity^15^; in fact, hippocampal structural alterations may affect episodic memory from childhood to early adulthood^16^. Furthermore, based on hippocampal subfield volumes, smaller left dentate gyrus volume has been related to reduced visual working memory in preterm children born between 25 and 34 weeks of gestation without brain pathology^17^.

There is a lack of research that focuses on neuropsychological outcomes in well-characterized samples in terms of their clinical manifestations (low- vs. high-risk preterm samples), which allows the effect of low and high-risk prematurity per se on cognition to be researched. Studies conferring neonatal brain damage following preterm birth have mainly observed impairments in global neuropsychological performance by providing a measure such as the intelligence quotient (IQ)^18^. For all these reasons, the purpose of this MRI study is to consider the long-term effects that high- and low-risk prematurity may have on the hippocampus of preterm children and their associations with memory performance. Four hypotheses have been proposed. First, high-risk preterm children with GM-IVH will have lower memory scores compared to their low-risk preterm and full-term peers in childhood. Second, those suffering from GM-IVH will evidence reduced hippocampal volumes in comparison to low-risk preterm and full-term groups. Third, hippocampal volumetric reductions might be related to memory performance in high-risk preterm children with GM-IVH. And fourth, the lower the GA, the higher the probability of maturing alterations during development^19^, which may result in low-risk preterm children without GM-IVH having decreased hippocampal volumetric values and poorer memory performance in contrast to their full-term peers.

## Results

The first analysis showed that a number of variables did not follow a normal distribution: (1) the high-risk preterm sample with GM-IVH: GA (Kolmogorov–Smirnov (K–S) = 0.25 *p* = 0.009), birth weight (BW) (K–S = 0.22 *p* = 0.03), A1 (K–S = 0.23 *p* = 0.03), and right hippocampus (K–S = 0.22 *p* = 0.03); (2) the low-risk preterm sample without GM-IVH: age (K–S = 0.28 *p* < 0.001), GA (K–S = 0.22 *p* = 0.02), internalizing problems (K–S = 0.23 *p* = 0.006), A1 (K–S = 0.22 *p* = 0.02), and A4 (K–S = 0.23 *p* = 0.008); and (3) the full-term sample: age (K–S = 0.29 *p* < 0.001), GA (K–S = 0.29 *p* < 0.001), internalizing problems (K–S = 0.22 *p* = 0.01), A1 (K–S = 0.25 *p* = 0.002), A3 (K–S = 0.20 *p* = 0.03), A5 (K–S = 0.22 *p* = 0.01), delayed recall (K–S = 0.22 *p* = 0.009), and right dentate gyrus (K–S = 0.23 *p* = 0.003).

Details of neonatal, clinical and sociodemographic variables are provided in Tables 1 and 2. As expected, there were significant differences between the two preterm groups in neonatal variables (GA and BW) in comparison to the full-term group. Besides, high-risk preterm-born children with GM-IVH showed greater immaturity (lower GA and BW) in both variables in relation to the low-risk preterm group without GM-IVH. No statistically significant differences were found in sex, age, handedness, or parental education among groups. Behavioral results showed no significant differences between groups with regard to internalizing, externalizing and total emotional-behavioral problems.

**Table 1:** Clinical data of high- and low-risk preterm samples.

	High-risk preterm n = 16	Percentage (%)	Low-risk preterm n = 20	Percentage (%)
Chorioamnionitis^a^	6/7	85.71	2/18	11.11
Antenatal steroids^a^	3/4	75	12/18	66.67
Apgar score < 6 at fifth minute^a^	4/14	28.57	0/18	0
Mechanical ventilation > 14 days^a^	3/13	23.08	0/18	0
Vaginal delivery^a^	8/15	53.33	5/18	27.78
Shunt	3/16	18.75	0/20	0
Seizures^a^	2/9	22.22	0/20	0
IUGR	2/16	12.5	2/20	10
SGA	3/16	18.75	5/20	25
**GM-IVH grades**				
Grade II	8	50		
Grade III	6	37.5		
Grade IV	2	12.5		

IUGR: intrauterine growth restriction; SGA: small for gestational age; and GM-IVH: germinal matrix-intraventricular hemorrhage.

^a^Available data for chorioamnionitis: 7 High-risk preterm and 18 Low-risk preterm; antenatal steroids: 4 High-risk preterm and 18 Low-risk preterm; Apgar score < 6 at fifth minute: 14 High-risk preterm and 18 Low-risk preterm; mechanical ventilation > 14 days: 13 High-risk preterm and 18 Low-risk preterm; vaginal delivery: 15 High-risk preterm and 18 Low-risk preterm; and seizures: 9 High-risk preterm and 20 Low-risk preterm.

**Table 2:** Neonatal, sociodemographic and emotional-behavioral variables.

	High-risk preterm n = 16 Mean ± SD	Low-risk preterm n = 20 Mean ± SD	Full-term n = 22 Mean ± SD	Statistics (*p*)
**Neonatal data**				
GA. wk [range]	28.00 ± 2.71 [25–36]	32.50 ± 1.36 [30–34]	39.50 ± 1.01 [38–41]	*H* = 47.62 (< .001)*
BW. g	1184.50 ± 559.18	1754.25 ± 451.55	3391.55 ± 356.96	*H* = 43.40 (< .001)*
**Sociodemographic data**				
Gender. male/female	7/9	11/9	14/8	*X*^2^ = 1.48 (.48)
Age. yo [range]	9.56 ± 2.31 [6–14]	9.30 ± 0.66 [8–10]	9.27 ± 0.63 [8–10]	*H* = 0.07 (.97)
Handedness right-handed/left-handed	13/3	18/2	22/0	*X*^2^ = 4.21 (.12)
Parental education low/intermediate/high	4/8/4	4/4/12	3/4/15	*X*^2^ = 8.16 (.09)
**Behavioral variables**				
Internalizing problems^a^	6.89 ± 5.35	8.99 ± 6.12	7.81 ± 4.72	*F* = 0.77 (.52)
Externalizing problems^a^	7.16 ± 6.61	9.36 ± 5.97	9.35 ± 5.61	*F* = 0.50 (.69)
Total behavioral problems^a^	22.99 ± 13.06	25.47 ± 14.66	24.70 ± 14.66	*F* = 0.24 (.87)

SD: standard deviation; GA: gestational age; wk: weeks; BW: birth weight; g: grams; yo: years; *K*: Kruskal–Wallis test; *X*^2^: Chi-square test; and *F*: Snedecor's F distribution. *Statistically significant differences between High-risk preterm < Low-risk preterm < Full-term.

^a^Available data for internalizing problems, externalizing problems and total problems: 16 GM-IVH-preterm, 20 low-risk preterm and 21 term children.

Regarding cognitive performance, as shown in Table 3, while mean Full Intelligence Quotient (FIQ) was within normal limits in both preterm groups, it was significantly lower compared to full-term children. Similarly, perceptual reasoning and verbal comprehension indexes showed significantly lower scores in both preterm born groups in comparison to their full-term peers. For their part, high-risk preterm children with GM-IVH obtained significantly lower scores in processing speed in relation to the full-term group. Memory performance was established by a composite score (see “Methods” section) with a Cronbach’s alpha coefficient of 0.85. All items from the Rey Auditory Verbal Learning Test, except for A1 and A4, as well as learning, delayed recall and the memory index, significantly differed between high-risk preterm sample with GM-IVH and both low-risk preterm and full-term groups; poorer performance was evidenced by those considered to be high-risk preterm children with GM-IVH. Conversely, A1 and A4 were significantly different among high-risk preterm children with GM-IVH evidencing lower scores in contrast to their full-term peers.

**Table 3:** Full intelligence quotient and learning-memory differences.

	High-risk preterm n = 16 Mean ± SD			Low-risk preterm n = 20 Mean ± SD	Full-term n = 22 Mean ± SD	*F-Snedecor statistic* (*p*)	$\eta_{p}^{2}$
**Wechsler intelligence scale for children IV**							
Verbal Comprehension	106.3 ± 13.29			107.25 ± 15.15	123.73 ± 19.04	7.38 (.001)*	.21
Perceptual Reasoning	94.9 ± 14.52			101.10 ± 13.59	115.64 ± 16.65	9.76 (< .001)*	.26
Working Memory	102.6 ± 13.90			107.60 ± 15.24	108.50 ± 16.25	0.77 (.46)	.03
Processing Speed	98.9 ± 13.02			107.25 ± 14.49	114.50 ± 8.85	7.62 (.001)**	.22
FIQ	99.7 ± 14.55			105.75 ± 13.76	121.91 ± 15.39	12.13 (< .001)*	.31
**Rey auditory verbal learning test (covariate according to age)**							
A1	4.84 ± 1.26			5.46 ± 1.28	6.02 ± 1.20	3.30 (.027)**	.16
A2	6.92 ± 2.16			8.73 ± 1.98	8.49 ± 1.77	4.38 (.008)***	.20
A3	8.45 ± 3.05			10.69 ± 2.08	10.78 ± 1.75	5.98 (.001)***	.25
A4	10.04 ± 3.47			11.60 ± 2.31	12.11 ± 1.46	5.44 (.002)**	.23
A5	10.79 ± 3.05			12.68 ± 1.69	12.90 ± 1.08	5.39 (.003)***	.23
Learning	41.03 ± 11.22			49.15 ± 7.93	50.30 ± 5.35	7.39 (< .001)***	.29
Delayed Recall	7.99 ± 4.14			10.97 ± 2.09	11.40 ± 1.50	6.00 (.001)***	.25
Memory	-0.75 ± 1.23			0.21 ± 0.76	0.35 ± 0.48	7.67 (< .001)***	.30

SD: standard deviation; FIQ: full intelligence quotient; WISC-IV: Wechsler Intelligence Scale for Children IV; RAVLT: Rey Auditory Verbal Learning Test; A1, A2, A3, A4 and A5: RAVLT trials; and $\eta_{p}^{2}$: partial eta squared.

*Statistically significant differences between High-risk preterm < Full-term and Low-risk preterm < Full-term.

**Statistically significant differences between High-risk preterm < Full-term.

***Statistically significant differences between High-risk preterm < Full-term and High-risk preterm < Low-risk preterm.

Global bilateral hippocampal volumes and grouped hippocampal subfield volumes (see “Methods” section), as presented in Table 4, were found to be significantly smaller in the high-risk preterm sample with GM-IVH compared to both low-risk preterm and full-term groups after Bonferroni correction for multiple comparisons was applied (*p* = 0.006). In other words, left and right global hippocampus, bilateral subiculum, bilateral CA-field and right dentate gyrus were found to be smaller in the high-risk preterm sample with GM-IVH. Nonetheless, the left dentate gyrus was only significantly different among high-risk preterm children with GM-IVH and the low-risk preterm group without GM-IVH (*p* < 0.001), with the high-risk preterm group with GM-IVH evidencing smaller values. All these results showed large effect sizes.

**Table 4:** Left/right hippocampus and grouped hippocampal subfield volumetric differences.

		High-risk preterm n = 16 Mean ± SD	Low-risk preterm n = 20 Mean ± SD	Full-term n = 22 Mean ± SD	*F-Snedecor Statistic* (*p*)		$\eta_{p}^{2}$
**Hippocampus (mm**^**3**^**) (covariate according to eTIV, age and presence of shunt valve)**							
Left Hippocampus		2,971.73 ± 579.95	3,459.27 ± 390.23	3,399.56 ± 271.96	13.00 (< .001)*		.56
Right Hippocampus		2,974.33 ± 424.70	3,352.35 ± 382.10	3,402.27 ± 288.50	26.53 (< .001)*		.72
**Grouped hippocampal subfields (mm**^**3**^**) (covariate according to eTIV, age and presence of shunt valve)**							
Left Subiculum		253.85 ± 62.63	301.99 ± 25.09	325.08 ± 31.07	10.73 (< .001)*		.51
Right Subiculum		241.49 ± 42.45	280.87 ± 29.85	299.92 ± 30.72	14.82 (< .001)*		.59
Left CA-field		1582.24 ± 322.00	1853.45 ± 231.12	1803.92 ± 156.22	12.74 (< .001)*		.55
Right CA-field		1606.23 ± 231.57	1812.39 ± 229.66	1841.89 ± 169.12	21.49 (< .001)*		.67
Left Dentate Gyrus		498.84 ± 86.81	567.66 ± 86.26	547.14 ± 53.92	13.38 (< .001)**		.56
Right Dentate Gyrus		509.74 ± 77.95	545.44 ± 77.38	545.16 ± 69.63	15.27 (< .001)*		.60

SD: standard deviation; mm^3^: cubic millimeter; and $\eta_{p}^{2}$: partial eta squared.

*Statistically significant differences between High-risk preterm < Full-term and High-risk preterm < Low-risk preterm.

**Statistically significant differences between High-risk preterm < Low-risk preterm. The global bilateral hippocampal volumes and grouped hippocampal subfield volumes shown in bold are those that remained significant after Bonferroni correction was applied for multiple comparisons (*p* = .006).

Attention should be drawn to the fact that the presence of a neonatal brain injury such as GM-IVH explained the variance in memory and neuroimaging variables (*p* < 0.05), while none of them were explained by GA. Furthermore, the interaction of both variables also explained the variance in left hippocampus (β = -3.60 *p* = 0.008 ∆R^2^ = 0.09), left CA-field (β = − 3.80 *p* = 0.006 ∆R^2^ = 0.10), and left dentate gyrus (β = − 2.99 *p* = 0.04 ∆R^2^ = 0.06).

**Figure 1:**
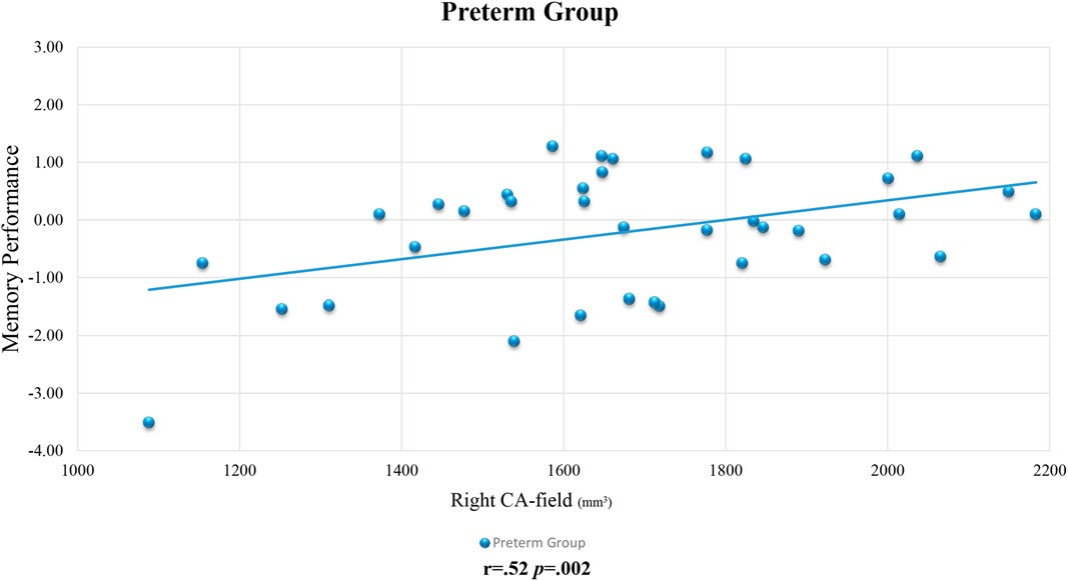
Correlation between the right CA-field and memory performance in preterm-born children. Note: mm^3^: cubic millimeter. Details of Pearson correlation coefficient (r) and *p*-corrected value are provided. Significant results were obtained between the right CA-field volume and memory performance after Bonferroni correction for multiple comparisons was applied (*p* = .006). Unstandardized residuals were calculated and used for display purposes (adjusted according to age and eTIV).

With reference to correlations in both preterm groups independently, none of the correlations between global and regional hippocampal volumetric measures and memory remained significant after Bonferroni correction for multiple comparisons was applied. As for the correlations in preterm and full-term groups, a significant correlation was observed between the right CA-field and memory performance in preterm-born children (see Fig. 1), remaining significant following Bonferroni correction for multiple comparisons (r = 0.52 *p-*corrected = 0.002). No statistically significant correlations were found in the full-term group.

As far as interaction analyses between high- and low-risk preterm children are concerned, significant differences were only found in the left subiculum correlation coefficients (*F* = 4.81 *p* = 0.04). No statistically significant interactions were found between preterm and full-term groups.

## Discussion

By including well-characterized high- and low-risk preterm groups, this study highlights the existence of different cognitive profiles in prematurity during childhood. Reduced hippocampal volumes were found only in those born prematurely, which reaffirms a biological susceptibility in preterm birth, leading to greater vulnerability in the hippocampus due to adverse pre-perinatal factors^13,14^. Moreover, memory performance was associated with the right CA-field volume only in those born prematurely. This structure–function relation between abnormal hippocampal growth and memory performance has previously been found in very low BW preterm adults with perinatal morbidity^20^. However, as is already known^21,22^, memory performance may not always be explained by hippocampal volumetric differences, since this is not the only structure that might be involved.

Our findings indicated lower scores in FIQ, verbal comprehension, and perceptual reasoning in high- and low-risk preterm-born children. Even though their cognitive performance was within normal range, preterm infants with or without neonatal brain damage continued to fall behind full-term children in several domains associated with intellectual functioning^7^, as well as in academic attainment^23^. Research identified lower scores in processing speed only in preterm children with GM-IVH. Processing speed deficits following preterm birth are strong predictors of executive function, inattentive behavior and academic achievement^24–26^; in fact, this cognitive domain has also been found to be affected in preterm toddlers without neurological abnormalities^27^. However, in this study, processing speed measurement required the involvement of motor abilities which, after a brain injury such as GM-IVH, is compromised and impacts overall processing speed performance^28,29^.

Regarding memory, results highlighted the adverse effect of being preterm with GM-IVH on memory skills, which had already been noted in heterogeneous preterm samples^30^. Furthermore, in this study, the worst learning and delayed recall scores were evidenced in a homogeneous sample of high-risk schooled preterm children with GM-IVH. However, at a different period of development and in the absence of neurologic abnormalities, similar outcomes were also evidenced by preterm toddlers; specifically, deficits in recall, immediate recognition and encoding speed^27^. Interestingly, in the case of the first immediate recall condition (A1), which might entail less involvement of the hippocampus compared to the following trials due to accumulated memory load, high-risk preterm children with GM-IVH also obtained lower scores than their full-term peers. This result stresses the fact that preterm children with atypical brain development have difficulty in retaining relevant information even over a short time interval, which might go beyond the hippocampal volumetric reductions described in this population, as other authors have suggested in preterm toddlers with subependymal or mild intraventricular hemorrhages^31^, and developmental amnesia^32^. Nevertheless, there was also found to be differentiation in A4 between high-risk preterm children with GM-IVH and their full-term peers. Finally, low-risk preterm born children without GM-IVH did not obtain lower scores in memory performance compared to full-term children. Therefore, as other authors have also demonstrated, memory may not be influenced by preterm birth in those considered to be at a lower risk of neurological abnormalities in childhood^33^, and adulthood^34^. In fact, although hippocampal growth may be affected due to prematurity, this might not imply memory impairments, as has also been shown in heterogeneous preterm children^35,36^.

In general, our results are in line with previous MRI analyses that reported reduced hippocampal volumes in heterogeneous preterm samples^16^. In fact, smaller hippocampal volumes have been detected in preterm infants with brain injury as well as with several perinatal events at 2 years of age^12,37^. Similar results were found in this study, since both global and regional hippocampal volumetric reductions were also observed in high-risk prematurity with GM-IVH in childhood. Regarding hippocampal laterality effects, our study found smaller volumes in both left and right hippocampal structures among GM-IVH children, as has already been reported by other authors, with a volumetric reduction of 15.6% in the right and 12.1% in the left hippocampus^8^. The hippocampal abnormalities in this study, apart from being bilateral, were also predominantly right-side; this is the hippocampus side that has been related to memory in young adults with very low BW^38^. Nevertheless, other studies have found a more symmetrical hippocampus^39^ and greater left predominance^15^, possibly due to altered WM development.

Furthermore, the hippocampal CA-field, which is particularly vulnerable to ischemia^40^, appears to be smaller in those suffering from a neonatal brain injury such as GM-IVH. Indeed, the right CA-field volume proved to be positively related to memory performance in preterm-born children, remaining significant after Bonferroni correction. According to Aanes et al.^17^, children with very low BW evidenced a weak correlation between CA-field volumes and number of days on mechanical ventilation. As a consequence, children with very low BW with the most immature lung function may have a greater likelihood of suffering from hypoxia–ischemia in the neonatal period, influencing the development of the CA-field. Nevertheless, among those who spent more than 14 days with mechanical ventilation in our study, there were only 3 children with GM-IVH and none without GM-IVH. On the other hand, the right dentate gyrus appear to be smaller in those suffering from a neonatal brain injury such as GM-IVH compared to both low-risk preterm and full-term groups; while left dentate gyrus differed from high-risk (GM-IVH) and low-risk preterm children. This structure has been stated as being susceptible to both physical and psychological stress, causing apoptosis and deficient neurogenesis. In fact, stress also impairs the impact of other neurological insults commonly encountered in prematurity such as hypoxia and ischemia^41^. Hence, these mechanisms might trigger reduced dentate gyrus volumes seen in the high-risk preterm group with GM-IVH. Finally, being aware that the subiculum is also vulnerable to hypoxic-ischemic episodes^42^, subiculum lower volumetric values were only found in those considered high-risk preterm children with GM-IVH. In fact, correlation coefficient differences were found in the left subiculum between high- and low-risk preterm children. Thus, in line with Aanes et al.^17^, the typical growth experienced by this structure might be negatively influenced by particular clinical conditions seen in preterm newborns.

Our results are consistent with the review by Nosarti and Froudist-Walsh^16^, which suggested that common brain injuries given after very preterm birth can affect common development of different memory networks. The current study found a robust relationship between the right CA-field volume and memory performance among preterm-born children, even in those without GM-IVH. Preterm hippocampal asymmetry has been evidenced as being smaller than in full-term infants^39^, and so disproportionate growth of the right hippocampus may give rise to atypical development. Indeed, abnormalities of hippocampal rightward asymmetry displayed in very preterm infants were related to memory alterations^39^—in other words, a more right lateralized hippocampal activation is a sign of a more mature brain, leading to better memory performance^43^. However, there would seem to be more severe long-lasting prevailing neurodevelopment alterations in preterm newborns with brain injury, resulting in numerous cognitive and behavioral dysfunctions^44^, even in adulthood^45^. Therefore, abnormalities in the hippocampal structure–function association might be more closely related to preterm birth alongside brain growth, as has already been observed in preterm children and adults with very low BW^17,38^.

Some limitations of the current study were the total sample size, the sample size of each group and also the wide age range included (6–16 years old), although our analyses were controlled according to age. The small sample size compels us to treat the results deriving from this study with caution. Therefore, further studies are needed in order to provide more evidence regarding hippocampal volumetric values and their association with memory performance in larger preterm samples that are followed longitudinally. Nevertheless, the study has a well-characterized sample since it differentiates between those preterm children with and without brain injury (i.e. with and without GM-IVH), this being deemed necessary when interpreting the possible effects of prematurity according to its clinical manifestations in brain structures and cognitive outcomes. In addition to the limited statistical power, the small sample size is also behind the inability to assess whether hippocampal volume and memory performance may be different when the different grades proposed by Papile et al^46^ are taken into consideration. Apparently, the more severe the grade, the greater the risk for perinatal morbidities and, specifically, the more negative long-lasting cognitive consequences will be^4^. In fact, despite high-risk preterm children commonly have a lower GA than their low-risk preterm peers without GM-IVH, the present study has proven that GM-IVH better explains memory and hippocampal volumetric differences after preterm birth. Furthermore, MRI images were obtained at different locations, even though the same scanner model and MRI parameters were used. Actually, to our knowledge, this is the first study that has found a structural–functional relationship between memory performance and the right CA-field in a well-characterized preterm sample in terms of their clinical manifestations by using a 3-T scanner with a 1 mm^3^ isotropic voxel. Furthermore, our study applied rigorous standards to ensure appropriate comparable groups, taking not only neonatal data but also parental education, and emotional-behavioral scores into consideration.

Another disadvantage is that the cross-sectional nature of our study did not make it possible to research into whether neurodevelopmental outcomes in memory due to reduced hippocampal volumes persist, worsen or improve over time. Feasibly, cognitive outcomes over the first few years of life reflect young adults’ performance, as displayed in preterm born young adults with very low BW^20^. In fact, the impact of very preterm birth on hippocampal volume and memory performance continues up to young adulthood and is related to the degree of neonatal brain injury^47,48^. Likewise, our volumetric analyses were limited to the hippocampus, although further structures may also be affected by neonatal brain injury in prematurity, since they are connected both anatomically and functionally. Finally, as memory can be divided into different subtypes, specific assessments of each one in prematurity such as visual memory may provide future considerations in the development of suitable interventions^32^.

A noteworthy contribution of our work is that memory function alterations and hippocampal volumetric reductions were shown in a sample of schooled preterm children with GM-IVH. In fact, understanding the scheme in which memory deficits arise in prematurity increases the chances of identifying children considered to be at a higher risk at an early stage. Moreover, despite the fact that hippocampal-memory associations were not found in high- and low-risk preterm children independently, a strong relationship between the right CA-field and memory was found only in preterm-born children (remaining significant after Bonferroni correction). Nevertheless, neuroanatomical substrates linked to memory are certainly complex, and so hippocampal volumetric differences per se may not completely elucidate memory performance^36^. In fact, according to Tamnes et al.^49^ specific hippocampal subfield volumes and developmental patterns may also be related to general cognition in healthy adolescents. Hence, more research is needed in homogeneous preterm samples in order to gain further insight into possible compensation mechanisms related to everyday cognitive functioning.

## Methods

### Study participants

A sample of preterm children considered to be at higher risk of developing neurodevelopmental alterations with a history of GM-IVH [henceforth the high-risk preterm group with GM-IVH] was recruited between March and November 2011. This sample belonged to a cohort that was followed longitudinally at the Vall d’Hebron University Hospital (Barcelona, Spain). The inclusion criteria for this study were: (1) preterm birth with a GA < 37 weeks of gestation; (2) having a neonatal diagnosis of GM-IVH grades II to IV in brain ultrasound imaging according to Papile classification^46^; and (3) age at time of evaluation of between 6 to 16 years old. The exclusion criteria involved being diagnosed with neonatal periventricular leukomalacia or any other perinatal cerebral insult, neurosensory alterations, a shunt valve non-compatible with the MRI scanner, or a FIQ ≤ 70. From the initial 107 children that were born at the Vall d’Hebron University Hospital with a neonatal diagnosis of GM-IVH, 24 were excluded due to periventricular leukomalacia (n = 13) or neurosensory alterations (n = 11). Eighty-three participants were initially invited to participate in the study, 50 of whom were not available to complete the assessment, and 6 of whom refused to take part in the study. Therefore, twenty-seven children were evaluated, although 1 of them was excluded since GM-IVH diagnosis could not be confirmed by clinical records, 3 had a GA > 37 weeks, 3 were left out because they had an FIQ score of ≤ 70 according to current evaluation and 1 participant’s GA data could not be retrieved from clinical records. Lastly, for the purpose of this study, 3 participants were excluded because T1 weighted images were not obtained (n = 1) or due to movement artifacts (n = 2). The resulting sample consisted of 16 high-risk preterm children with GM-IVH, most of whom were extremely preterm or very preterm children (i.e. eight extremely preterm and six very preterm). No differences were found in sex, age, handedness, FIQ or GM-IVH grades between the high-risk preterm participants included (n = 16) and excluded (n = 11). However, as expected, differences were found in both GA and BW, since three excluded children had a GA > 37 weeks and a BW > 3000 g.

The other two groups belonged to a cohort that had been previously studied at the University of Barcelona (Barcelona, Spain), with their characteristics already having been published elsewhere^6,10^. Since we were interested in isolating the specific impact of GM-IVH on brain measurements, a group of preterm children considered to be at low risk of evidencing major disabilities was included [known henceforth as the low-risk preterm group without GM-IVH]. The inclusion criteria were: (1) a GA of between 30 to 34 weeks’ gestation; (2) no brain pathology as assessed by neonatal cranial US; (3) BW of under 2500 g; (4) Apgar score at 5 min > 7; (5) no substantial neonatal morbidity; and (6) age at time of evaluation of between 6 to 16 years old. Seventy-six preterm children were found to meet these criteria following a search at the Hospital Clinic (Barcelona, Spain). Nevertheless, updated addresses were not available for 36 preterm children, and parents of 19 others declined to take part. Finally, abnormalities in the MRI findings (i.e. giant arachnoid cyst) of one child determined its exclusion. Hence, this low-risk preterm group without GM-IVH comprised 20 children, most of whom were being moderate or late preterm children (i.e. seven moderate preterm and seven late preterm). In addition, a full-term group was included with the following inclusion criteria: (1) GA ≥ 37 weeks; (2) BW higher than 2500 g; and (3) age at time of evaluation of between 6 to 16 years old. Twenty-three full-term children who were mostly classmates of the preterm children assessed were included in the present study. However, due to abnormalities in MRI findings (i.e. venous vascular malformation) one child was excluded, meaning that the full-term group comprised 22 participants. The exclusion criteria for both low-risk preterm and full-term groups were: experiencing any MRI counter-indications, a history of acquired brain injury, cerebral palsy or other neurological impairment, or a FIQ ≤ 70.

Parental education was calculated by the maximum number of years’ education completed by the parents, classified into low (primary education or junior secondary vocational education), intermediate (general or senior secondary education) and high (higher vocational education or university)^50^.

Ethical authorization for the study was approved by both the Ethics Committee of the Vall d’Hebron University Hospital and the Ethics Committee of the University of Barcelona. All participants’ parents gave written informed consent, and all methods were pursued in accordance with relevant guidelines and regulations.

### Neuropsychological assessment

To assess memory performance, all participants were evaluated using the *Rey Auditory Verbal Learning Test*^51^, which was chosen because of its recognized sensitivity to declarative memory impairments. Participants were asked to repeat a 15-word list over five trials (A1-A5), with the following measurements being obtained: (a) learning, sum of the immediate recall of the 15-word list learnt over five trials; and (b) delayed recall of the number of words after 20 min of interference.

The *Wechsler Intelligence Scale for Children IV*^52^ was used to measure intellectual ability by assessing the following four indexes: Verbal Comprehension, Perceptual Reasoning, Working Memory, and Processing Speed. Furthermore, FIQ was able to be obtained by adding all subtests together.

Emotional-behavioral information was collected via the *Child Behavior Checklist*^53^, which was used to measure children’s emotional-behavioral functioning based on parents’ reports. It is made up of eight subscales and some of them were grouped into higher order factors, namely internalizing and externalizing problems. The sum of the above-mentioned eight subscales provided the total score.

### MRI images

3-dimensional MRI datasets were obtained for the GM-IVH group at the *Unitat de Diagnòstic per la Imatge* (Vall d'Hebron University Hospital, Barcelona, Spain) and for the low-risk preterm and full-term groups using the same 3-T scanner and the same sequence parameters at the *Diagnostic Center for Imaging* (Hospital Clínic, Barcelona, Spain). MRI images were obtained from a TIM TRIO 3-T machine (Siemens, Erlangen, Germany), and an MPRAGE sequence was acquired in sagittal orientation (TR/TE = 2300/2.98 ms, TI = 900 ms, 256 × 256 matrix, flip angle 9º, 1 mm^3^ isotropic voxel). To identify GM-IVH lesions in the high-risk preterm sample, T2-weighted images were also acquired in axial orientation (TR/TE = 5150/99 ms; 512 × 307 matrix, flip angle 120º, slice thickness 5 mm with a 1.5 intersection gap), as well as fluid-attenuated inversion recovery images (axial orientation, TR/TE = 9040/85 ms, TI = 2500, 256 × 156 acquisition matrix, flip angle 150º, slice thickness 5 mm with a 1.5 intersection gap). For low-risk preterm and full-term subjects, T2-weighted images (axial orientation, TR/TE = 5533/88 ms, 122 × 122 matrix, flip angle 90º, slice thickness 2 mm, gap = 0.6 mm) were also acquired. Even though T2-weighted images were obtained using different parameters (GM-IVH group vs. low-risk preterm and full-term groups), these images were merely used for clinical estimation of WM intensities. All MRI data were checked by two pediatric neuroradiologists (E.V. and I.D.) to provide updated GM-IVH information and detect any brain alteration.

Prior to processing, images were also checked for movement and scanner artifacts. T1-weighted images were analyzed using FreeSurfer (https://surfer.nmr.mgh.harvard.edu/) (version v6.0.0) in order to obtain hippocampal volumes, and hippocampus segmentations were made of 3D T1-weigthed structural MRI scans^54^. Processing of T1 high-resolution images included several procedures: intensity non-uniformity correction, skull stripping, affine transformation to MNI template, intensity normalization, removal of non-brain tissue, linear and nonlinear transformations to a probabilistic brain atlas and labelling of subcortical/allocortical structures. Spatial localization priors were used to determine the correct label per each single voxel^55^.

**Figure 2:**
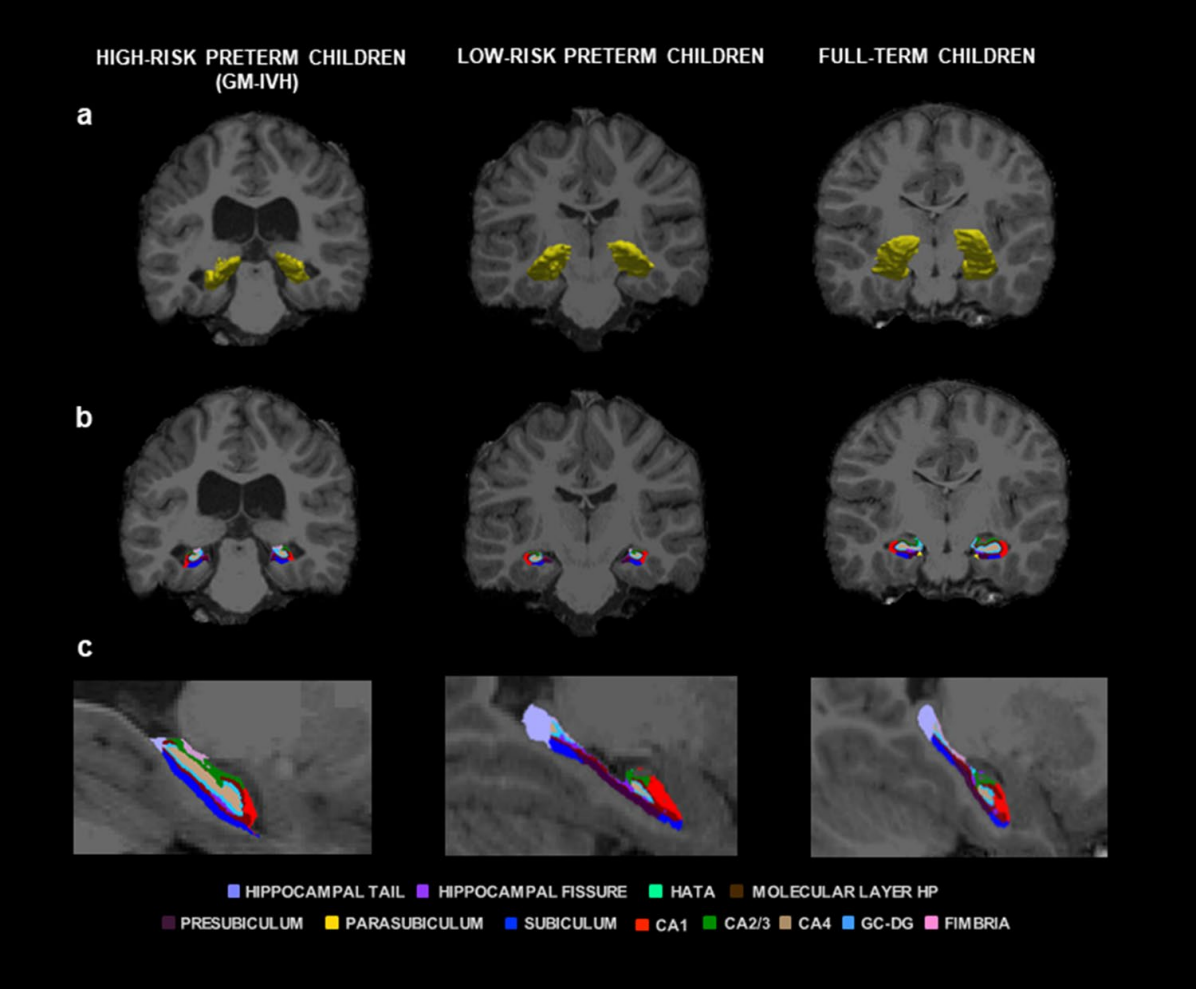
Right and left hippocampus and hippocampal subfield volumes. Coronal (**a**,**b**) and sagittal (**c**) views showing volumetric segmentation among groups in right and left hippocampus and hippocampal subfields. In accordance with radiological convention, the left side is shown on the right of the image for each view. CA: Cornu Ammonis, GC–DG: granule cell layer of dentate gyrus, HATA: hippocampal-amygdalar transition area; HP: hippocampus; and GM-IVH: germinal matrix-intraventricular hemorrhage. Coronal coordinate (**a**,**b**): x: -205.43, y: -11.00, z: -127.00; and sagittal coordinate (**c**): x: 2.50, y: 140.00, z: -50.05.

T1-weighted images were processed using a hippocampal subfield segmentation package, Freesurfer hippocampal-subfields-T1 command (i.e. using the Freesurfer hippocampal subfield pipeline)^56^, with left and right hippocampal values were obtained. Moreover, the hippocampus was segmented into 12 subfields for each hemisphere: hippocampal tail, subiculum, presubiculum, parasubiculum, Cornu Ammonis 1 (CA1), CA2/3, CA4, granule cell layer of dentate gyrus (GC–DG), molecular layer hippocampus (HP), fimbria, hippocampal-amygdalar transition area (HATA), and hippocampal fissure (see Fig. 2). In line with previous authors who have used this type of segmentation in preterm samples^17^, and since our segmentations were based only on T1-weighted images and certain hippocampal subfields are considered less reliable because of their size^56^, three grouped subfield volumes were created for each hippocampal hemisphere: CA-field (CA1 + CA2/3 + molecular layer HP + subiculum); Dentate gyrus (GC-DG + CA4); and Subiculum (presubiculum). This reclassification is more in accordance with proposals put forward by Mueller et al.^57^ and other automated segmentation methods^58^. All hippocampal volumetric values are expressed in mm^3^.

### Statistical analysis

Normal distribution of the data was assessed using the K–S test, with tests being run on raw scores for normally distributed data and on ranked dependent variables for not normally distributed data. A composite score was calculated to create a memory index with learning and delayed recall scores; to do so all raw cognitive scores were converted into z-values, and Cronbach’s alpha coefficient was used to determine the internal consistency of this composite score.

The Kruskal–Wallis test was used to analyze differences in neonatal data (GA and BW) and age at time of evaluation. Furthermore, the Chi-squared test was required to assess differences in the following qualitative sociodemographic characteristics: sex, handedness and parental education. Moreover, a multivariate analysis of covariance was used to compare neuropsychological functioning and neuroanatomical volumetric values in the hippocampus (i.e. left and right hippocampus provided by Freesurfer and grouped hippocampal subfields) among the three groups. Covariates were: (1) age for memory scores; and (2) total intracranial volume (eTIV), age and whether or not they had a ventriculoperitoneal shunt for MRI volumetric comparisons. Additional analyses were conducted to regress the effect of sex on memory and hippocampal measures and to add scanner site as covariate; however, both results failed to differ after multivariate analyses of covariance were undertaken among the three groups. Partial eta squared was used to calculate the effect sizes of those comparisons. Bonferroni’s post-hoc test was used to assess differences between groups, and Bonferroni corrected *p* value for significance was also calculated for global bilateral hippocampal volumes and the three bilateral grouped hippocampal subfield volumes (*p* = 0.05/8 = 0.006). Besides, in order to ascertain to what extent our results might be explained by neonatal brain injury, GA or the interaction of both variables, regression analyses were conducted on all memory and hippocampal volumetric variables.

Partial correlations (adjusted for eTIV and age) were performed between the memory index and global (left/right) as well as regional hippocampal volumes grouped into three subfields for each hemisphere (i.e. CA-field, dentate gyrus and subiculum) in both preterm groups independently and also in preterm and full-term groups. In order to correct for multiple comparison purposes, Bonferroni corrected *p* value for significance was calculated (*p* = 0.05/8 = 0.006). Likewise, interaction analyses were conducted to ascertain whether memory-hippocampal associations were statistically different between high- and low-risk preterm children as well as between preterm and full-term groups. For all preceding analyses, the SPSS version 26 was used, and significance level was set at 0.05.

### Ethics approval

Ethical authorization for the study was approved by both the Ethics Committee of the Vall d’Hebron University Hospital and the Ethics Committee of the University of Barcelona.

## Consent for publication

All participants’ parents gave written informed consent to participate and for publication.

## Data Availability

The data sets and analysis of current study are available from the corresponding author upon reasonable request.
